# Crystal structure of 7,7-dimethyl-6-methyl­idenetri­cyclo­[6.2.1.0^1,5^]undecane-2-carb­oxy­lic acid

**DOI:** 10.1107/S2056989014028254

**Published:** 2015-01-10

**Authors:** Noureddine Beghidja, Samir Benayache, Fadila Benayache, David W. Knight, Benson M. Kariuki

**Affiliations:** a Unité de recherche VARENBIOMOL, Constantine 1 University, Constantine 25000, Algeria; b School of Chemistry, Cardiff University, Main Building, Park Place, Cardiff CF10 3AT, UK

**Keywords:** crystal structure, *inula graveolens*, hydrogen bonding

## Abstract

In the title compound, C_15_H_22_O_2_, both five-membered rings display an envelope conformation whereas the six-membered ring displays a chair conformation. In the crystal, pairs of O—H⋯O hydrogen bonds between carb­oxy­lic groups link mol­ecules, related by a twofold rotation axis, into supra­molecular dimers.

## Related literature   

For background to the title compound, which was extracted from the air-dried aerial parts of *inula graveolens* see: Chiappini & Fardella (1980 ▸); Rustaiyan *et al.* (1987 ▸). For related structures, see: Turner *et al.* (1980 ▸); Harlow & Simonsen (1977 ▸); Dastlik *et al.* (1992 ▸).
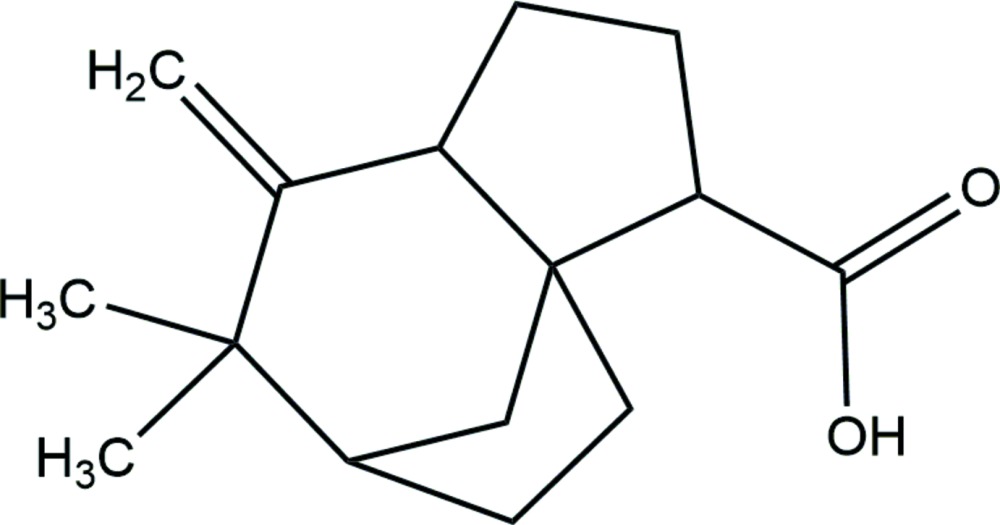


## Experimental   

### Crystal data  

C_15_H_22_O_2_
*M* *_r_* = 234.33
Orthorhombic, 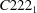

*a* = 7.6400 (3) Å
*b* = 16.1700 (5) Å
*c* = 21.3406 (9) Å
*V* = 2636.39 (17) Å^3^
*Z* = 8
Mo *K*α radiation
μ = 0.08 mm^−1^
*T* = 150 K
0.30 × 0.18 × 0.04 mm

### Data collection  

Nonius KappaCCD diffractometer
Absorption correction: multi-scan (*DENZO*/*SCALEPACK*; Otwinowski & Minor, 1997 ▸) *T*_min_ = 0.978, *T*_max_ = 0.997
8814 measured reflections
2978 independent reflections
2327 reflections with *I* > 2σ(*I*)
*R*_int_ = 0.052

### Refinement  

*R*[*F* ^2^ > 2σ(*F* ^2^)] = 0.047
*wR*(*F* ^2^) = 0.103
*S* = 1.07
2978 reflections
157 parameters
H-atom parameters constrained
Δρ_max_ = 0.15 e Å^−3^
Δρ_min_ = −0.15 e Å^−3^

### 

Data collection: *COLLECT* (Nonius, 2000 ▸); cell refinement: *HKL* *SCALEPACK* (Otwinowski & Minor 1997 ▸); data reduction: *HKL* *DENZO* (Otwinowski & Minor 1997 ▸) and *SCALEPACK*; program(s) used to solve structure: *SIR92* (Altomare *et al.*, 1992 ▸); program(s) used to refine structure: *SHELXL97* (Sheldrick, 2008 ▸); molecular graphics: *ORTEP99* for Windows (Farrugia, 2012 ▸); software used to prepare material for publication: *WinGX* publication routines (Farrugia, 2012 ▸) and *CHEMDRAW Ultra* (Cambridge Soft, 2001 ▸).

## Supplementary Material

Crystal structure: contains datablock(s) I, New_Global_Publ_Block. DOI: 10.1107/S2056989014028254/xu5833sup1.cif

Structure factors: contains datablock(s) I. DOI: 10.1107/S2056989014028254/xu5833Isup2.hkl

Supporting information file. DOI: 10.1107/S2056989014028254/xu5833Isup3.cml

**Fig. 1.**
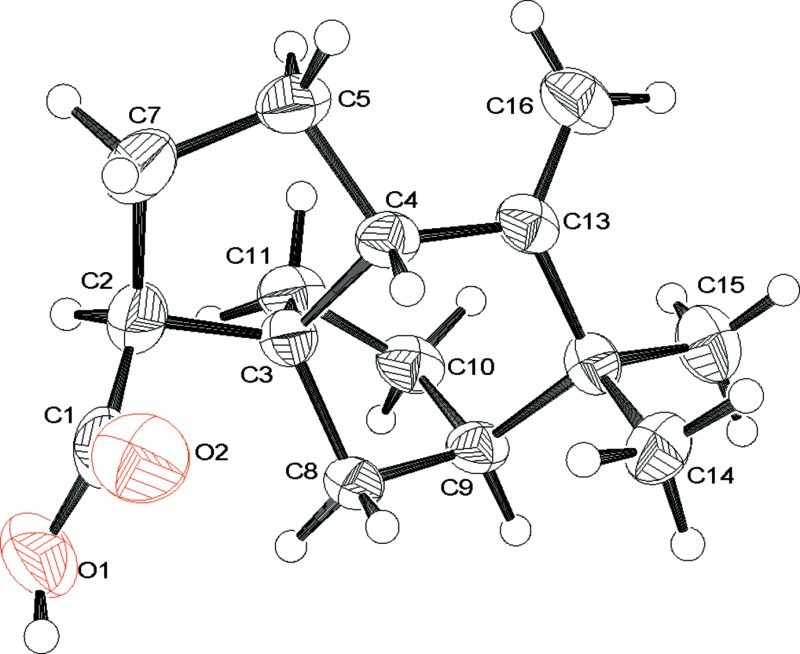
A molecule showing atom labels and 50% probability displacement ellipsoids for non-H atoms.

. DOI: 10.1107/S2056989014028254/xu5833fig1.tif
A mol­ecule showing atom labels and 50% probability displacement ellipsoids for non-H atoms.

**Fig. 2.**
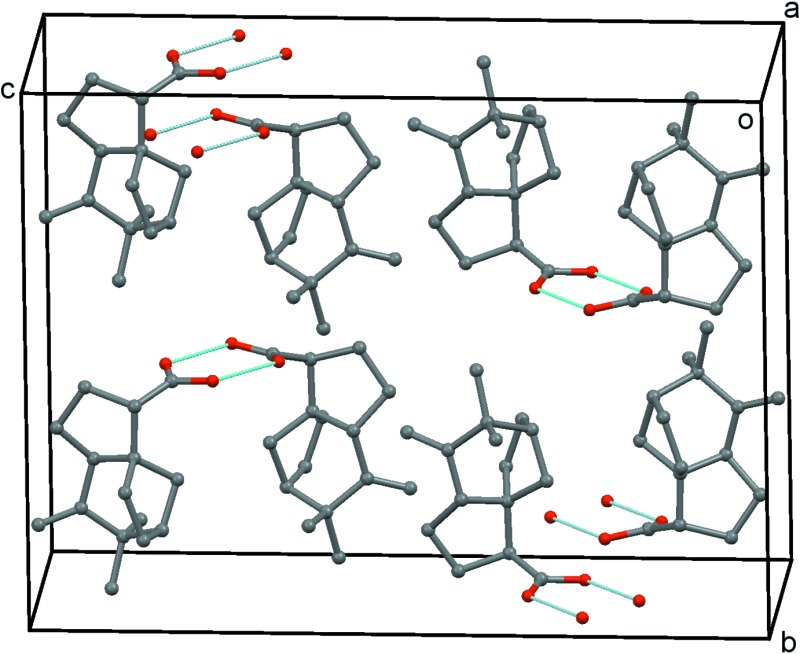
Crystal packing in the structure with H atoms omitted and hydrogen bonds shown as dotted lines.

. DOI: 10.1107/S2056989014028254/xu5833fig2.tif
Crystal packing in the structure with H atoms omitted and hydrogen bonds shown as dotted lines.

CCDC reference: 1041493

Additional supporting information:  crystallographic information; 3D view; checkCIF report

## Figures and Tables

**Table 1:** Hydrogen-bond geometry (, )

*D*H*A*	*D*H	H*A*	*D* *A*	*D*H*A*
O1H1O2^i^	0.84	1.81	2.646(3)	174

Symmetry code: (i) 
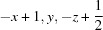
.

## supplementary crystallographic information

### S1. Comment

*Inula graveolens* have consistently been the subject of research interest (Chiappini & Fardella, 1980; Rustaiyan *et al.*, 1987). Our interest is the extracts from aerial parts of Algerian species such as stems, flowers and leaves. The asymmetric unit of the crystal structure consists of a single molecule (Fig. 1). Both five-membered rings display an envelope conformation (with C4 and C8 as the flap atoms) whereas the six-membered ring displays a chair conformation.

The structure consists of pairs of molecules linked by the classic dimeric carbocylic acid hydrogen bonding interaction (Fig 2). Structures of some related compounds have been reported (Turner *et al.*, 1980; Harlow & Simonsen, 1977; Dastlik *et al.*, 1992).

### S2. Experimental

The air-dried aerial parts of *inula graveolens* (500 g) were extracted with acetone/Et_2_O (1:1) at room temperature. The solution was filtered off and concentrated under reduced pressure to give a pale yellow gum (9 g). The gum was subjected to successive column chromatography (silica gel) and TLC (silica gel, PF254). Eleven fractions were obtained. Fraction 9 gave a material which crystallized as colourless crystals with a melting point of 450 K.

### S3. Refinement

H atoms were positioned geometrically and refined using a riding model with *U*_iso_(H) constrained to be 1.2 times *U*_eq_ for the atom it is bonded to (except for methyl groups where it was 1.5 times with free rotation about the C—C bond).

### Crystal data

**Table:**

C_15_H_22_O_2_	*D*_x_ = 1.181 Mg m^−^^3^
*M**_r_* = 234.33	Mo *K*α radiation, λ = 0.71073 Å
Orthorhombic, *C*222_1_	Cell parameters from 2327 reflections
*a* = 7.6400 (3) Å	θ = 2.7–27.4°
*b* = 16.1700 (5) Å	µ = 0.08 mm^−^^1^
*c* = 21.3406 (9) Å	*T* = 150 K
*V* = 2636.39 (17) Å^3^	Plate, colourless
*Z* = 8	0.30 × 0.18 × 0.04 mm
*F*(000) = 1024	

### Data collection

**Table:**

Nonius KappaCCD diffractometer	2978 independent reflections
Radiation source: fine-focus sealed tube	2327 reflections with *I* > 2σ(*I*)
Graphite monochromator	*R*_int_ = 0.052
CCD slices, ω and phi scans	θ_max_ = 27.4°, θ_min_ = 2.7°
Absorption correction: multi-scan (*DENZO*/*SCALEPACK*; Otwinowski & Minor, 1997)	*h* = −9→8
*T*_min_ = 0.978, *T*_max_ = 0.997	*k* = −20→20
8814 measured reflections	*l* = −27→27

### Refinement

**Table:**

Refinement on *F*^2^	Primary atom site location: structure-invariant direct methods
Least-squares matrix: full	Secondary atom site location: difference Fourier map
*R*[*F*^2^ > 2σ(*F*^2^)] = 0.047	Hydrogen site location: inferred from neighbouring sites
*wR*(*F*^2^) = 0.103	H-atom parameters constrained
*S* = 1.07	*w* = 1/[σ^2^(*F*_o_^2^) + (0.0392*P*)^2^ + 0.6557*P*] where *P* = (*F*_o_^2^ + 2*F*_c_^2^)/3
2978 reflections	(Δ/σ)_max_ = 0.001
157 parameters	Δρ_max_ = 0.15 e Å^−^^3^
0 restraints	Δρ_min_ = −0.15 e Å^−^^3^

### Special details

**Table:**

Geometry. All s.u.'s (except the s.u. in the dihedral angle between two l.s. planes) are estimated using the full covariance matrix. The cell s.u.'s are taken into account individually in the estimation of s.u.'s in distances, angles and torsion angles; correlations between s.u.'s in cell parameters are only used when they are defined by crystal symmetry. An approximate (isotropic) treatment of cell s.u.'s is used for estimating s.u.'s involving l.s. planes.
Refinement. Refinement of *F*^2^ against ALL reflections. The weighted *R*-factor *wR* and goodness of fit *S* are based on *F*^2^, conventional *R*-factors *R* are based on *F*, with *F* set to zero for negative *F*^2^. The threshold expression of *F*^2^ > 2σ(*F*^2^) is used only for calculating *R*-factors(gt) *etc*. and is not relevant to the choice of reflections for refinement. *R*-factors based on *F*^2^ are statistically about twice as large as those based on *F*, and *R*- factors based on ALL data will be even larger.

### Fractional atomic coordinates and isotropic or equivalent isotropic displacement parameters (Å^2^)

**Table:**

	*x*	*y*	*z*	*U*_iso_*/*U*_eq_	
C1	0.3233 (3)	0.41972 (11)	0.18712 (10)	0.0496 (5)	
C2	0.1890 (3)	0.39139 (12)	0.14061 (9)	0.0469 (5)	
H2	0.0705	0.4118	0.1532	0.056*	
C3	0.1900 (2)	0.29473 (10)	0.13886 (8)	0.0351 (4)	
C4	0.3048 (2)	0.27610 (11)	0.08041 (7)	0.0331 (4)	
H4	0.4293	0.2879	0.0918	0.040*	
C5	0.2468 (3)	0.34157 (11)	0.03307 (8)	0.0419 (4)	
H5A	0.3350	0.3485	−0.0005	0.050*	
H5B	0.1330	0.3268	0.0139	0.050*	
C7	0.2312 (3)	0.41978 (13)	0.07268 (9)	0.0586 (6)	
H7A	0.1365	0.4556	0.0564	0.070*	
H7B	0.3423	0.4513	0.0718	0.070*	
C8	0.2606 (3)	0.24567 (10)	0.19461 (7)	0.0355 (4)	
H8A	0.2004	0.2609	0.2340	0.043*	
H8B	0.3884	0.2534	0.1997	0.043*	
C9	0.2161 (2)	0.15726 (11)	0.17434 (7)	0.0348 (4)	
H9	0.2277	0.1182	0.2104	0.042*	
C10	0.0230 (2)	0.16680 (12)	0.15525 (8)	0.0415 (4)	
H10A	−0.0550	0.1569	0.1915	0.050*	
H10B	−0.0075	0.1273	0.1215	0.050*	
C11	0.0058 (3)	0.25713 (12)	0.13183 (9)	0.0422 (5)	
H11A	−0.0323	0.2584	0.0875	0.051*	
H11B	−0.0801	0.2880	0.1575	0.051*	
C12	0.3311 (2)	0.12798 (11)	0.11790 (8)	0.0345 (4)	
C13	0.2924 (2)	0.18576 (11)	0.06300 (7)	0.0330 (4)	
C14	0.5274 (2)	0.13066 (12)	0.13538 (9)	0.0428 (4)	
H14A	0.5965	0.1063	0.1014	0.064*	
H14B	0.5465	0.0992	0.1740	0.064*	
H14C	0.5634	0.1882	0.1418	0.064*	
C15	0.2877 (3)	0.03737 (11)	0.10297 (9)	0.0504 (5)	
H15A	0.1635	0.0326	0.0922	0.076*	
H15B	0.3129	0.0030	0.1397	0.076*	
H15C	0.3591	0.0187	0.0675	0.076*	
C16	0.2421 (2)	0.16103 (13)	0.00671 (8)	0.0462 (5)	
H16A	0.2123	0.2007	−0.0244	0.055*	
H16B	0.2358	0.1037	−0.0026	0.055*	
O1	0.2608 (2)	0.42962 (10)	0.24438 (7)	0.0639 (4)	
H1	0.3442	0.4330	0.2699	0.096*	
O2	0.4779 (2)	0.42873 (9)	0.17426 (7)	0.0606 (4)	

### Atomic displacement parameters (Å^2^)

**Table:**

	*U*^11^	*U*^22^	*U*^33^	*U*^12^	*U*^13^	*U*^23^
C1	0.0665 (16)	0.0313 (9)	0.0511 (12)	0.0092 (10)	0.0070 (11)	−0.0002 (9)
C2	0.0534 (13)	0.0419 (10)	0.0455 (11)	0.0164 (9)	0.0036 (10)	0.0009 (9)
C3	0.0345 (10)	0.0406 (10)	0.0302 (8)	0.0087 (8)	0.0013 (8)	−0.0016 (7)
C4	0.0282 (9)	0.0425 (9)	0.0286 (8)	0.0027 (8)	0.0001 (7)	0.0016 (7)
C5	0.0381 (11)	0.0547 (11)	0.0329 (8)	0.0078 (9)	0.0026 (8)	0.0100 (8)
C7	0.0752 (17)	0.0499 (12)	0.0507 (11)	0.0192 (11)	0.0035 (11)	0.0137 (9)
C8	0.0405 (10)	0.0413 (10)	0.0248 (8)	0.0060 (8)	0.0006 (7)	−0.0018 (7)
C9	0.0345 (10)	0.0433 (10)	0.0265 (8)	0.0004 (8)	0.0025 (7)	0.0029 (7)
C10	0.0338 (10)	0.0583 (12)	0.0326 (9)	−0.0032 (9)	0.0092 (8)	0.0004 (8)
C11	0.0310 (10)	0.0622 (12)	0.0335 (9)	0.0088 (9)	0.0067 (8)	0.0038 (8)
C12	0.0348 (10)	0.0347 (9)	0.0341 (9)	0.0009 (8)	0.0049 (8)	−0.0032 (7)
C13	0.0237 (9)	0.0458 (10)	0.0296 (8)	−0.0001 (7)	0.0063 (7)	−0.0030 (7)
C14	0.0360 (11)	0.0432 (10)	0.0492 (11)	0.0064 (8)	−0.0006 (8)	0.0046 (9)
C15	0.0534 (14)	0.0422 (11)	0.0558 (12)	−0.0047 (9)	0.0107 (11)	−0.0075 (9)
C16	0.0405 (12)	0.0621 (12)	0.0361 (9)	−0.0022 (10)	0.0062 (9)	−0.0090 (9)
O1	0.0667 (11)	0.0715 (9)	0.0536 (8)	0.0157 (8)	0.0057 (8)	−0.0223 (8)
O2	0.0667 (11)	0.0592 (9)	0.0560 (9)	−0.0160 (8)	0.0063 (8)	0.0078 (7)

### Geometric parameters (Å, º)

**Table:**

C1—O2	1.221 (3)	C9—C12	1.564 (2)
C1—O1	1.322 (2)	C9—H9	1.0000
C1—C2	1.499 (3)	C10—C11	1.549 (3)
C2—C7	1.555 (3)	C10—H10A	0.9900
C2—C3	1.564 (2)	C10—H10B	0.9900
C2—H2	1.0000	C11—H11A	0.9900
C3—C8	1.529 (2)	C11—H11B	0.9900
C3—C11	1.540 (3)	C12—C13	1.527 (2)
C3—C4	1.554 (2)	C12—C15	1.536 (2)
C4—C13	1.510 (2)	C12—C14	1.546 (3)
C4—C5	1.529 (2)	C13—C16	1.323 (2)
C4—H4	1.0000	C14—H14A	0.9800
C5—C7	1.526 (3)	C14—H14B	0.9800
C5—H5A	0.9900	C14—H14C	0.9800
C5—H5B	0.9900	C15—H15A	0.9800
C7—H7A	0.9900	C15—H15B	0.9800
C7—H7B	0.9900	C15—H15C	0.9800
C8—C9	1.532 (2)	C16—H16A	0.9500
C8—H8A	0.9900	C16—H16B	0.9500
C8—H8B	0.9900	O1—H1	0.8400
C9—C10	1.538 (3)		
			
O2—C1—O1	122.9 (2)	C10—C9—C12	111.40 (14)
O2—C1—C2	123.34 (19)	C8—C9—H9	110.6
O1—C1—C2	113.7 (2)	C10—C9—H9	110.6
C1—C2—C7	112.66 (19)	C12—C9—H9	110.6
C1—C2—C3	108.54 (15)	C9—C10—C11	105.13 (15)
C7—C2—C3	105.77 (15)	C9—C10—H10A	110.7
C1—C2—H2	109.9	C11—C10—H10A	110.7
C7—C2—H2	109.9	C9—C10—H10B	110.7
C3—C2—H2	109.9	C11—C10—H10B	110.7
C8—C3—C11	101.16 (14)	H10A—C10—H10B	108.8
C8—C3—C4	108.95 (13)	C3—C11—C10	105.28 (14)
C11—C3—C4	111.16 (14)	C3—C11—H11A	110.7
C8—C3—C2	120.12 (14)	C10—C11—H11A	110.7
C11—C3—C2	113.12 (15)	C3—C11—H11B	110.7
C4—C3—C2	102.45 (14)	C10—C11—H11B	110.7
C13—C4—C5	119.27 (15)	H11A—C11—H11B	108.8
C13—C4—C3	110.45 (14)	C13—C12—C15	112.50 (15)
C5—C4—C3	103.45 (13)	C13—C12—C14	110.84 (14)
C13—C4—H4	107.7	C15—C12—C14	106.63 (15)
C5—C4—H4	107.7	C13—C12—C9	107.26 (13)
C3—C4—H4	107.7	C15—C12—C9	109.12 (15)
C7—C5—C4	103.33 (14)	C14—C12—C9	110.51 (14)
C7—C5—H5A	111.1	C16—C13—C4	122.27 (17)
C4—C5—H5A	111.1	C16—C13—C12	124.60 (17)
C7—C5—H5B	111.1	C4—C13—C12	113.00 (14)
C4—C5—H5B	111.1	C12—C14—H14A	109.5
H5A—C5—H5B	109.1	C12—C14—H14B	109.5
C5—C7—C2	106.75 (16)	H14A—C14—H14B	109.5
C5—C7—H7A	110.4	C12—C14—H14C	109.5
C2—C7—H7A	110.4	H14A—C14—H14C	109.5
C5—C7—H7B	110.4	H14B—C14—H14C	109.5
C2—C7—H7B	110.4	C12—C15—H15A	109.5
H7A—C7—H7B	108.6	C12—C15—H15B	109.5
C3—C8—C9	100.72 (13)	H15A—C15—H15B	109.5
C3—C8—H8A	111.6	C12—C15—H15C	109.5
C9—C8—H8A	111.6	H15A—C15—H15C	109.5
C3—C8—H8B	111.6	H15B—C15—H15C	109.5
C9—C8—H8B	111.6	C13—C16—H16A	120.0
H8A—C8—H8B	109.4	C13—C16—H16B	120.0
C8—C9—C10	101.20 (14)	H16A—C16—H16B	120.0
C8—C9—C12	112.04 (14)	C1—O1—H1	109.5

### Hydrogen-bond geometry (Å, º)

**Table:**

*D*—H···*A*	*D*—H	H···*A*	*D*···*A*	*D*—H···*A*
O1—H1···O2^i^	0.84	1.81	2.646 (3)	174

Symmetry code: (i) −*x*+1, *y*, −*z*+1/2.
