# Supplementary figures and images for: Crystal structure of 7,7-dimethyl-6-methyl­idenetri­cyclo­[6.2.1.01,5]undecane-2-carb­oxy­lic acid
Source: Acta Crystallogr E Crystallogr Commun. 2015 Jan 10;71(Pt 2):o94. doi: 10.1107/S2056989014028254 (PMC4384579; doi:10.1107/S2056989014028254)

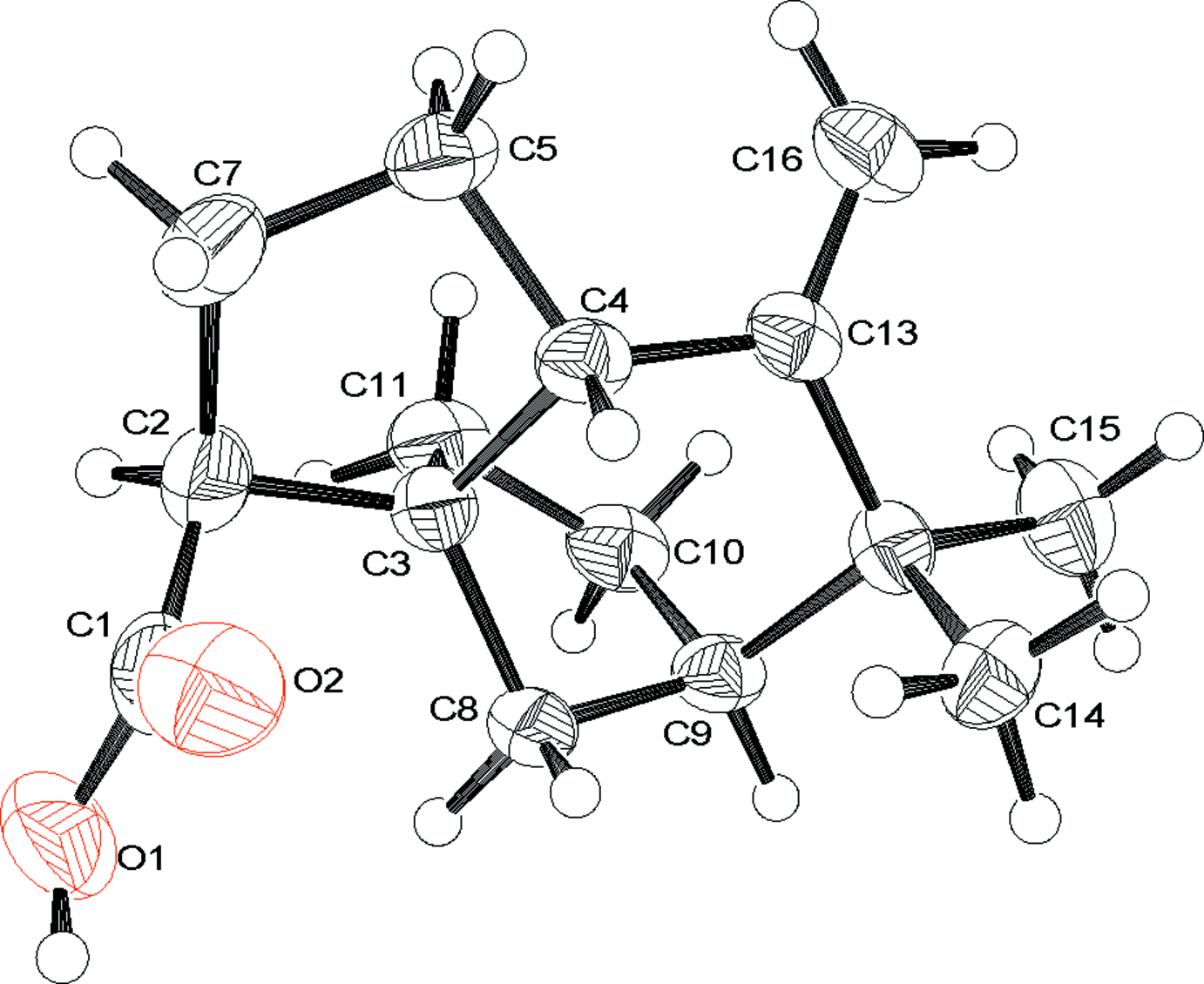

Supplement: Supplementary file 4 [file e-71-00o94-fig1.tif]

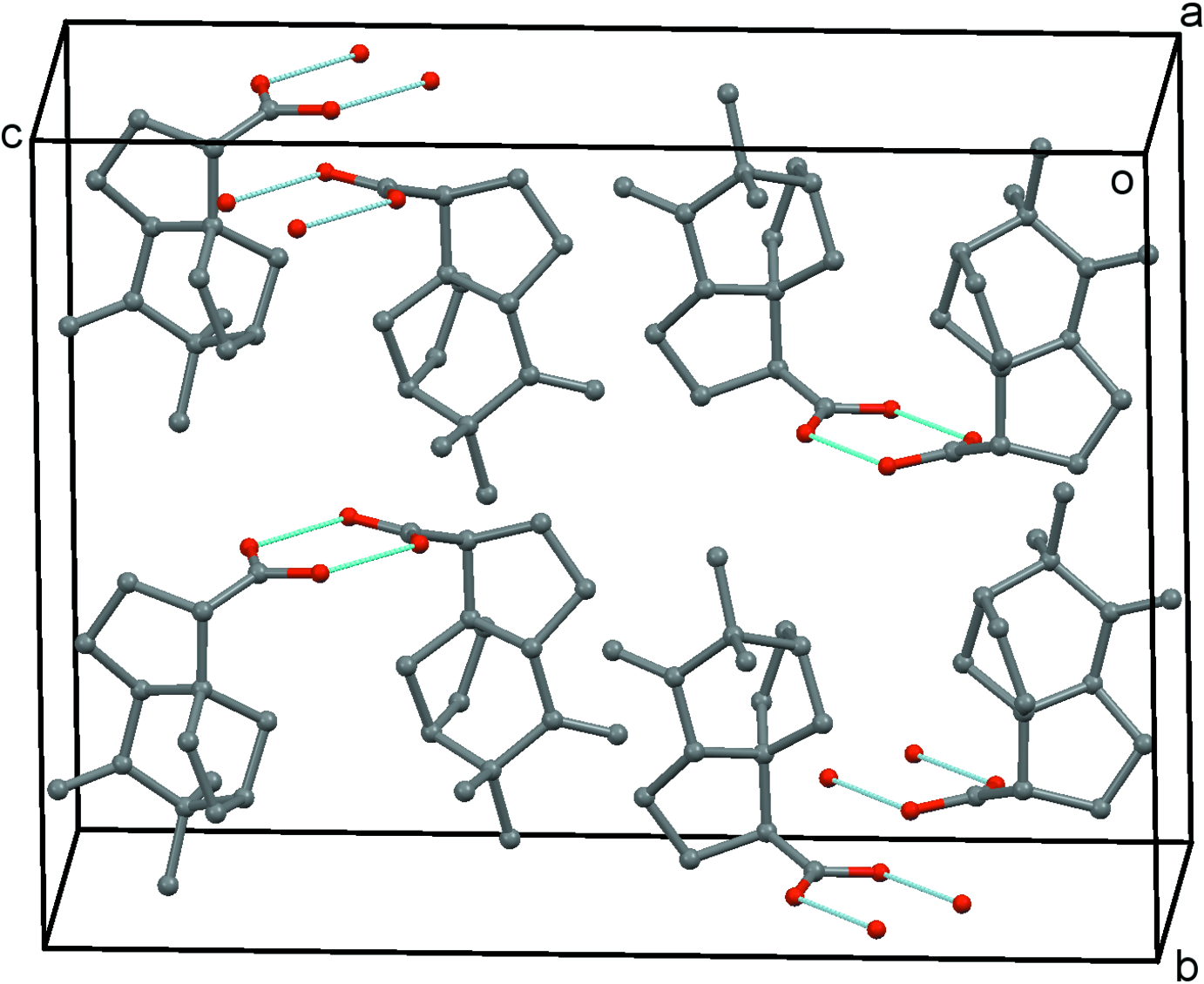

Supplement: Supplementary file 5 [file e-71-00o94-fig2.tif]
